# Retrograde aortic approach for atrial fibrillation ablation with a conventional 3‐D mapping catheter: A case report

**DOI:** 10.1002/ccr3.2075

**Published:** 2019-02-27

**Authors:** Christian Comandante, Po‐Cheng Chang, Yu‐Chang Huang, Chung‐Chuan Chou

**Affiliations:** ^1^ Division of Cardiology, Department of Medicine Chang Gung Memorial Hospital Linkou Taiwan; ^2^ Department of Electrophysiology Philippine Heart Center Manila Philippines; ^3^ Chang Gung University College of Medicine Taoyuan Taiwan

**Keywords:** ablation, atrial fibrillation, atrial septal defect, electroanatomic mapping, retrograde aortic approach

## Abstract

The presence of a Gore‐Tex patch can create difficulty in performing transeptal puncture for atrial septal defect patients underwent atrial fibrillation ablation. The maneuverability and stability of using manually operated catheters via retrograde aortic approach could be overcome by a large‐curved catheter to form a loop facilitating approachability to all parts of left atrium.

## 1 CASE REPORT

We report a patient with secundum‐type atrial septal defect postsurgical Gore‐Tex patch repair presenting with paroxysmal drug‐refractory atrial fibrillation (AF). Three‐dimensional (3‐D) electroanatomic mapping‐guided radiofrequency catheter ablation with conventional large‐curved irrigating ablation catheter via retrograde aortic approach was performed to create circumferential pulmonary veins isolation and linear lesions at left atrial roof and posterior wall successfully. Retrograde aortic approach via manually operated 3D‐electroanatomic mapping‐guided radiofrequency catheter ablation is a viable alternative when transeptal puncture has failed for AF ablation.

A 52‐year‐old female with prior surgical closure of a secundum‐type atrial septal defect (ASD) was referred to our hospital for symptomatic paroxysmal AF. She has hypertension for 5 years with regular medical control. AF episodes were sensed as chest discomfort that would last for 2‐3 hours. Flecainide and amiodarone had been given but AF paroxysms continued, and she was advised to undergo AF ablation. Preprocedure echocardiogram showed no residual interatrial shunt, a dilated left atrium (LA, anteroposterior diameter of 53 mm, Table 1), mild‐to‐moderate mitral regurgitation, and a normal left ventricular ejection fraction. Thyroid function tests and other laboratory data including lipid profile, glycohemoglobin, and electrolytes were all within normal range.

**Table 1:** Left atrial remodeling after radiofrequency catheter ablation

	LAD (mm)	LA_max _(mL)	LA_min _(mL)	LAEF (%)	LVEDD (mm)	LVESD (mm)	LVEF (%)	MR degree
Baseline	53	117	63	46	48	27	75	Mild‐moderate
1 mo	45	107	57	47	45	26	74	Mild‐moderate
3 mo	43	95	44	54	46	22	83	Mild
6 mo	43	87	34	61	43	24	76	Mild
12 mo	41	63	23	63	46	24	78	Mild
18 mo	38	56	25	55	45	24	77	Mild

LAD, LA dimension; LA_max_, maximal LA volume; LA_min_, minimal LA volume; LAEF, LA emptying fraction = (LA_max _− LA_min_)/LA_max_; LVEDD, left ventricular end diastolic dimension; LVESD, left ventricular end systolic dimension; LVEF, left ventricular ejection fraction; MR, mitral regurgitation.

**Figure 1:**
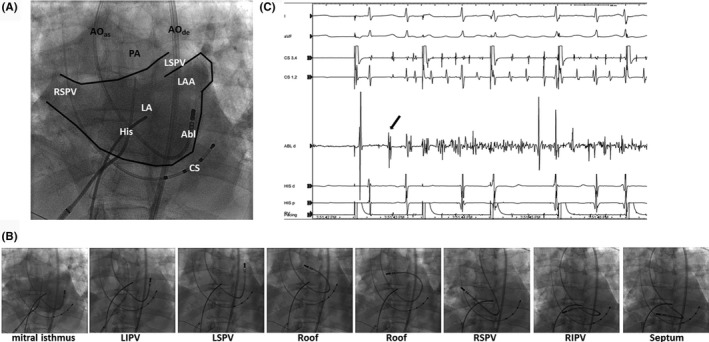
A, Angiogram of LA and PVs in the left anterior oblique 30° projection. B, Fluoroscopic images illustrate the maneuver of J‐curved mapping catheter in the LA. C, Endocardial tracings showed the earliest activation site was located at the ablation catheter near the left PVs (red arrow) during AF initiation. AO_as_, ascending aorta; AO_de_, descending aorta; Abl, catheter for ablation and mapping; CS, coronary sinus; LA, left atrium; LAA, left atrial appendage; LSPV, LIPV, RSPV, RIPV: left superior, inferior and right superior, posterior pulmonary veins, respectively

The procedure was performed under general anesthesia with 1%‐2% isoflurane. A 6F quadripolar diagnostic catheter was inserted into the coronary sinus through the right internal jugular vein, and a 6F decapolar diagnostic catheter was inserted through the right femoral vein and placed at the His to mark the inferior border of the aortic root. A Brockenbrough needle was advanced into the Mullins sheath (Medtronic, Minneapolis, MN, USA) via the right femoral vein, and transesophageal echocardiography (TEE)‐guided transeptal puncture was attempted. However, there was resistance in penetrating the Gore‐Tex patch to obtain transseptal access. After several failed attempts at transeptal puncture, we decided to convert to a retrograde aortic approach. A 3.5‐mm‐tip large‐curved (J curve) open‐irrigating catheter (Biosense Webster, Diamond Bar, CA, USA) was inserted through an 8.5F SL0 sheath (St Jude Medical, St Paul, MN, USA) in the femoral artery and maneuvered across the aortic and mitral valves to the LA. Next, 34 mL of contrast was injected at 15 mL/s through a Berman angiographic catheter (Teleflex, Morrisville, NC, USA) lodged into the main pulmonary artery to visualize the pulmonary veins (PV) and LA (Figure 1A).

**Figure 2:**
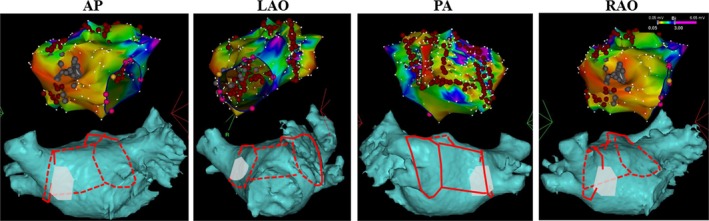
Three‐dimensional electroanatomic maps of the left atrium (LA, upper subpanels) illustrating the ablation lesions at anterior‐posterior (AP), left anterior oblique (LAO), posterior‐anterior (PA) and right anterior oblique (RAO) views. As seen from the red ablation points, the pulmonary veins had been encircled and additional ablation lines were located at the LA roof and posterior wall. Lower subpanels, corresponding CT images of the LA. Red solid and dotted lines indicate ablation lines on the front and rear side, respectively. Gray color zone indicates the location of Gore‐Tex patch

Heparin was used to maintain the activated clotting time at the level of 300 seconds. An electroanatomic map of the LA was constructed using a three‐dimensional (3‐D) mapping system (CARTO 3, Biosense Webster). The ablation catheter was manually manipulated to map the LA and PVs (Figure 1B). Spontaneous AF occurred with the earliest activation site near the left PVs (Figure 1C). Circumferential PV isolation and linear ablations at the LA roof and posterior wall were performed (Figure 2). Radiofrequency energy was continuously delivered with a power of 25‐30 W and irrigation rate of 17 mL/min, and a maximum temperature of 42°C (Stockert, Biosense Webster). PV isolation was confirmed by mapping the ablation lines around the PVs and assessing the absence of PV potentials. Since AF persisted after circumferential PV isolation and LA linear ablation, direct current cardioversion was performed to restore sinus rhythm. Cavo‐tricuspid isthmus ablation was carried out. The total procedure duration for LA mapping and ablation was 148 minutes, including the total radiofrequency delivery time of 37.5 minutes. The average power was 28 W. No adverse events were observed.

Follow‐up was conducted routinely at 1 week, 1 month, 3 months, 6 months, and 12 months and then every 6 months after ablation and whenever required because of the symptoms of AF. Serial 12‐lead electrocardiograms and 24‐hour Holter ambulatory electrocardiogram were recorded after ablation and when patients exhibited symptoms of palpitation.^1^ Flecainide was prescribed at the dosage of 100 mg bid during the first 3 months. As there was no recurrence of AF, the dosage was decreased to 100 mg/d for the next 3 months, and then discontinued thereafter. She remains free of recurrence of any atrial tachyarrhythmia during an 18‐month follow‐up. The serial echocardiograms showed reduction of LA dimension and volume, improvement of LA emptying fraction and mitral regurgitation (Table 1).

## 2 DISCUSSION

AF has been shown to persist and progress even after surgical repair of ASD, especially in older patients.^2^ AF ablation through a transeptal approach is safe and effective among patients who had undergone ASD patch closure.^3^ However, the presence of an intra‐atrial patch can create difficulty in performing a transeptal puncture, especially when a wide Gore‐Tex patch is used.^4^ In our case, TEE‐guided transeptal puncture was attempted but we encountered resistance in traversing the atrial septum. It has been demonstrated that retrograde aortic approach using remote magnetic navigation and a 3‐D mapping system is a feasible option to gain access to anatomic structure of the LA and perform AF ablation.^5^ In our case, we opted to perform AF ablation with a retrograde aortic approach using a conventional J‐curved open‐irrigating catheter and 3D‐electroanatomical mapping system. The procedure was successful in preventing AF recurrence with no complications.

Remote magnetic navigation‐guided retrograde aortic approach has been reported to be effective, safe, and fluoroscopy‐saving for complex LA ablation in patients with difficult transseptal access to the LA.^6^ However, remote magnetic navigation system may not always be available for AF ablation. In using conventional catheters for AF ablation via a retrograde aortic approach, the maneuverability and stability of catheters would be an issue.^5^ We did encounter interference from left ventricle contractions during ablation. To stabilize the catheter position, we used a large curve catheter to form a loop to facilitate approachability to all parts of the LA, and the target of local signals elimination could be achieved successfully. The other concern of a retrograde approach is the risk of aortic and mitral valves injury.^7^ Repeat echocardiograms performed after the procedure showed neither significant aortic regurgitation nor aggravation of mitral regurgitation.

To our knowledge, this is the first reported case of AF ablation through a retrograde aortic approach using a conventional ablation catheter.

## CONFLICT OF INTEREST

None declared.

## AUTHOR CONTRIBUTION

CC: involved in preparing and writing the paper. P‐CC and Y‐CH: involved in helping performing ablation procedures. C‐CC: was main operator of the ablation procedures, reviewing, and revising the paper as the corresponding author.
